# Acute effects of different warm-up duration on internal load and external load responses of soccer players in small sided games

**DOI:** 10.1186/s13102-025-01132-3

**Published:** 2025-04-09

**Authors:** Osman Yilmaz, Furkan Ozturk, Ladislav Batalik

**Affiliations:** 1 School of Physical Education and Sports, Osmaniye Korkut Ata University, Osmaniye, Turkey; 2 Graduate Education Institute, Tokat Gaziosmanpasa University, Tokat, Turkey; 3 Department of Physiotherapy and Rehabilitation, Faculty of Medicine, Masaryk University, Brno, Czech Republic; 4 Department of Rehabilitation, University Hospital Brno, Brno, Czech Republic; 5 Department of Public Health, Faculty of Medicine, Masaryk University, Brno, Czech Republic; 6 Rehabilitation Clinic, Faculty of Medicine, Masaryk University, Brno, Czech Republic

**Keywords:** Small-Sided Games, Warm-up duration, Psychophysiology, Technical skills

## Abstract

**Background:**

Soccer is a dynamic sport that involves high-intensity running, changes of direction, jumping and contact. Therefore, a proper warm-up duration is of great importance to optimize players'performance and minimize the risk of injury.

**Methods:**

This study examined the responses of amateur young 16 players (age = 17.00 ± 0.81 years; height = 177.38 ± 5.50 cm; weight = 64.50 ± 5.45 kg) 25 min (min), 15 min and 8 min warm-up duration in 4 v 4 small-sided games (SSGs) with mini-goal formats. Participants are assessed using the Participant Classification Framework, they are categorized under Tier 2: Trained/Developmental. The SSG interventions were randomly assigned to three training intervention groups. The features of SSG are determined as size; 25 × 32 m, bout; 4 × 4 min, resting; 4 min. Before the SSG, same protocol was applied at different times in all warm-ups. Warm-up protocols consisted of 13 sections. The intervention time in each section decreased parallel to the total 25 min, 15 min and 8 min warm-up times. The rating of perceived exertion (RPE), heart rate (HR) responses, distance covered and technical activities were consistently recorded during all SSG sessions. A one-way repeated-measures ANOVA was used to assess significant differences in performance among the different warm-up duration.

**Results:**

After the interventions, HR, total player load (TPL), successful passes (SP), unsuccessful passes (USP), interceptions and lost ball results demonstrated significant difference between the 25-min, 15-min and 8-min warm-up durations (*p* < 0.05). Total distance, velocity, RPE and enjoyment results showed no significant difference between the 25-min, 15-min and 8-min warm-up duration (*p* > 0.05). Results indicate that a 15-min warm-up duration provides an optimal balance between physiological and technical preparation, leading to improved HR responses, SP and interceptions compared to the 25-min and 8-min warm-ups. The 25-min warm-up decreased USP and lost ball occurrences compared to the 15-min and 8-min warm-ups. The 8-min warm-up resulted in a lower TPL, indicating reduced physiological demands.

**Conclusions:**

The 15-min warm-up duration emerged as an optimal protocol, offering a time-efficient approach that enhances both technical performance and physiological readiness while avoiding unnecessary fatigue. This finding provides practical implications for coaches and practitioners in designing warm-up routines that maximize match readiness without overexertion.

## Introduction

Soccer is a sport characterized by dynamic movements such as running, jumping, dribbling, and kicking [1]. Additionally, soccer players requirement moderate to high levels of aerobic and anaerobic power, as well as agility and a variety of tactical and technical skills, to improve their chances of success [2–4]. Small-sided games (SSGs) are a widely used training method designed to replicate match conditions, modifying game rules to optimize physical, technical, and tactical demands [5, 6]. SSGs allow players to develop energy systems and metabolic efficiency while engaging in real-game scenarios [7–9]. Given the importance of optimizing training efficiency, the structure and duration of warm-ups before SSGs and matches are essential for obtaining these requirements.

The purpose of a warm-up in soccer is to prepare the player for the demands of the training or match [10]. A sufficient and good warm-up increases the increase blood temperature, blood circulation, physiological response, motor performance, technical skill adaptation and cognitive conditioning required for the match or training, while also minimizing the risk of injury [11–15]. Traditional warm-up protocols typically consist of light to moderate-intensity exercises, which help increase flexibility, activate neural pathways, and improve reaction times [16–19].

The impact of warm-up protocols on acute performance has been extensively studied in team sports, highlighting their role in enhancing metabolic and neuromuscular efficiency [20]. A well-designed warm-up can stimulate muscle activation, increase conduction velocity, and improve oxygen uptake kinetics, all of which contribute to enhanced sprinting performance and reduced injury risk [13, 21, 22]. Additionally, raising core body temperature through warm-ups has been shown to enhance sprint performance, repeated sprint ability, and overall match preparedness [23–25].

Despite the known benefits of warm-ups, their optimal duration remains a subject of debate. In team sports, pre-match warm-ups typically last between 15 and 45-min (min), with an average duration of 30-min [10, 23]. However, prolonged warm-ups may unnecessarily extend total exertion time, leading to premature fatigue [26, 27]. Some studies suggest that shorter warm-ups (10–15-min) are sufficient to enhance performance while minimizing energy depletion [13]. Research also indicates that excessive warm-up duration can negatively affect performance due to increased perceived exertion and accumulated fatigue [26, 28].

Previous studies have explored the effects of various warm-up durations on physical performance, particularly in areas such as sprinting, jumping, agility, and perceived exertion. For example, research has compared 20-min and 10-min warm-up (^wup^) on sprint tests and perceived exertion in soccer players [29]. Other studies have analyzed the effects of 25-min^wup^, 15-min^wup^ and 8-min^wup^ on change of direction, vertical jump, and RPE in soccer players [24] and the impact of warm-up duration on neuromuscular and reactive strength indices in futsal players [20]. Additional research has investigated warm-up duration effects on muscle soreness, fatigue levels, sprint performance and agility in handball and rugby players [30, 31].

However, despite extensive research on warm-up durations and physical performance, few studies have examined their effects on actual in-game performance in soccer. SSGs are widely used in training because they replicate match conditions, promoting tactical and technical development while maintaining high-intensity physiological demands. Understanding how different warm-up durations impact SSG performance is crucial in designing effective pre-game and pre-training warm-up protocols.

### Study objective

This study aims to examine the impact of different warm-up durations (8-min, 15-min, and 25-min) on performance during 4 v 4 small-sided games. By analyzing both internal (heart rate, perceived exertion) and external (technical skills, movement metrics) load responses, this study seeks to provide practical insights for optimizing warm-up strategies in soccer.

## Materials and methods

### Participants

**Fig. 1:**
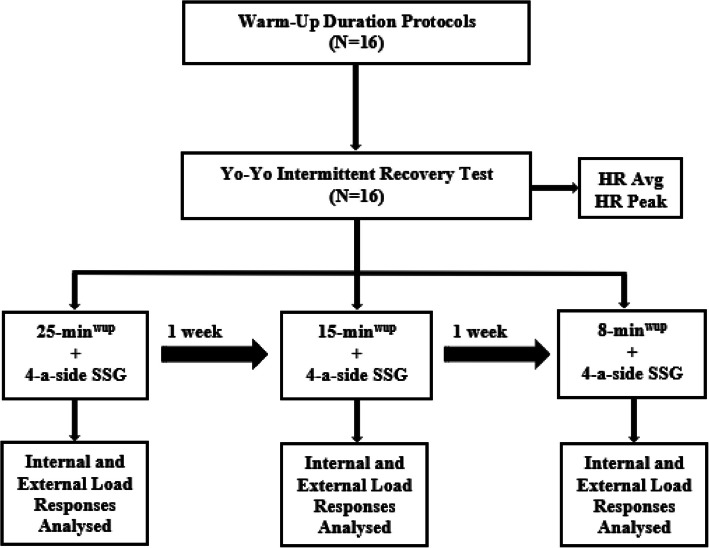
Study design

Young amateur 16 soccer players (age = 17.00 ± 0.81 years; height = 177.38 ± 5.50 cm; weight = 64.50 ± 5.45 kg) randomly played four bouts 4-a side SSG after 25-min^wup^, 15-min^wup^ and 8-min^wup^ duration protocols (Fig. 1). Choosing young amateur players encompasses a wider demographic in terms of applicability. Furthermore, when participants are assessed using the Participant Classification Framework, they are categorized under Tier 2: Trained/Developmental [32]. Participants are young amateur soccer players competing in local tournaments. They train regularly ∼3 times a week and play a match once a week. The research was conducted during the competition phase of the season, in the last three weeks of the second competition period. Before the study, participants performed three warm-up methods were familiarized on three different days. The players who did not have any physical or mental tiredness, injuries, pain, or illnesses before the study volunteered to take part. The participants were provided with verbal information on the contents of the study, as well as its techniques, procedures, advantages, and potential risks. The research was conducted in compliance with the Declaration of Helsinki and obtained clearance from the Ethics Committee of Osmaniye Korkut Ata University (31.05.2024-E.180447).

### Design

A cross-sectional observational study approach was employed to investigate the hypotheses. The research was conducted within three regular team training sessions, each of which was separated by one week. A warm-up protocol consisting of specified activities was completed by all sixteen players who took part in the study. The different warm-up duration protocols employed in the program established by Yanci et al. [24]. Table 1 presents information on warm-up protocols. Previous to the Small-Sided Games (SSG), participants'fitness was assessed using the Yo-Yo Intermittent Recovery Test Level- 1 to facilitate equal team assignment based on the results [33]. The Yo-Yo Intermittent Recovery Test is a valuable tool for measuring and monitoring fitness levels, particularly in sports characterized by intermittent exertion. This context conveys information regarding the current physical performance state of athletes. The peak heart rate response has been recorded as the maximum heart rate value in the YYIRTL- 1 test. Subsequent to the test, the estimated maximal oxygen consumption (VO2_max_) was computed utilizing the following formula (Estimated VO2_max_ = 36.4 + (0.0084 × covered distance in YYIRT- 1) [33]. The teams were determined equally according to these results. Both internal and external loads were recorded for all SSG. SSG were implemented as, 25-min^wup^ + 4-a-side, 15-min^wup^ + 4-a-side, 8-min^wup^ + 4-a-side with mini goals. These conditions were tested only once per participant due to practical constraints and to simulate real-game scenarios where teams follow a single warm-up protocol before matches. The order of different warm-up durations combined with SSG was determined by randomization (www.randomization.com). Random allocation sequence created by the authors. The features of SSG are determined as size; 25 × 32 m, number of bouts; 4, bouth duration; 4-min, resting duration; 4-min. Participants indicated their perceived exertion and enjoyment both between bouts and following each SSG session. Training sessions were conducted on a grass soccer surface at comparable times to reduce the impact of circadian rhythms (chronobiological characteristics) on the outcomes.

**Table 1:** Description of the characteristics of each of the warm-up duration

Content	Warm-up 25-min	Warm-up 15-min	Warm-up 8-min
Aerobic work and joint mobility (min)	0–3	0–2	0–1
Individual and collective technical exercises (min)	3–11	2–6	1–3
Free circulation with the ball (min)	3–5	2–3	1–1.30
Pass (min)	5–7	3–4	1.30–2
Ball driving (min)	7–9	4–5	2–2.30
Possession 4 vs.1 (min)	9–11	5–6	2.30–3
Static stretching (min)	11–13	6–7	3–3.30
Dynamic stretching (min)	13–14	7–8	3.30–4.30
Small sided game 4 vs. 4 (min)	14–16	8–9	4.30–5
Break (min)	16–17	9–10	5–5.30
Small sided game 4 vs. 4 (min)	17–19	10–11	5.30–6
Long passes (min)	19–23	11–13	6–7
Sprint starts (min)	23–25	13–15	7–8
**Total time (min)**	**25**	**15**	**8**

### Warm-up protocols

Yanci et al. [24] applied this warm-up protocol to semi-professional soccer players in their study. The standard warm-up procedure implemented by the team prior to official matches was utilized as a reference (25-min^wup^). The duration for each activity was proportionally decreased in both the 15-min^wup^ and 8-min^wup.^ The regimen encompassed aerobic activities, joint mobility exercises, individual and collective technical drills, static and dynamic stretching, two segments of a small-sided game including 4 vs. 4, long ball passes, and sprint beginnings. The comprehensive details of the warm-up protocols are given in Table 1.

### Measurements

#### Yo-Yo intermittent recovery test level 1

The YYIRTL- 1 is a dependable and acoustically regulated progressive test that involves consecutive 20-m runs back and forth between the starting, turning, and finishing lines. Participants were required to finish their shuttle runs within ten seconds, with brief periods of active recovery consisting of jogging or walking in between each shuttle run. It estimates an individual's maximum oxygen consumption (VO2 max), indicating their aerobic capacity and overall fitness level. The testing protocol generally consists of a uniform warm-up, succeeded by incremental shuttle runs till fatigue. The distance covered, or the level attained prior to fatigue, serves as an indicator of performance. The assessment was conducted in a natural grass field following the protocols proposed by Bangsbo et al. [33].

### Heart rate monitor and motion analysis system

The heart rate (HR) of the players during the games was monitored using the Polar V800, a device manufactured by Polar Electro Oy in Kempele, Finland. This equipment is capable of recording heart rate at one-second intervals and detecting pulse from the wrist. Moreover, it has the capability to ascertain the distance covered during the matches.

### Psychophysiological responses

The rate of perceived exertion (RPE) was utilized to determine the level of felt effort immediately following each session. A standardized question (How was the exercise and how did you feel exercise?) was employed to maintain consistency [34, 35]. Previous research established the validity and reliability of this scale for assessing effort intensity [36]. To eliminate any bias, players submitted their responses individually and were familiarized with the scale before answering, hence enhancing the dependability of their responses. The scale ranged from 0 to 10, with higher ratings indicating increased exertion [37]. Additionally, all participants were required to fill out an exercise enjoyment questionnaire, which utilizes a Likert scale ranging from 1 to 7 to assess the level of satisfaction experienced during physical activity. The validity of the scale as a measure of exercise enjoyment in Turkish adolescents and adult athletes has been confirmed by Raedeke [38] and Soylu et al. [39].

### Technical responses

High-resolution digital cameras, namely a Canon LEGRIA HF R806 from Tokyo, Japan, were utilized in order to capture video recordings of all SSGs. A highly skilled and experienced match and performance analysis coach then used the eAnalyze Soccer program (Espor Digital, Ankara, Turkey) to conduct an analysis of the technical activities that were carried out. The analysis encompasses successful passes, unsuccessful passes, interception and lost ball technical parameters. A successful pass is typically defined as one that reaches a teammate without interference from the opponent. An unsuccesful pass is typically defined as one directed toward the opponent rather than the player's teammate. Interception is defined as intruding in the opponent's pass and taking the ball from the adversary during a duel. Lost ball is considered as a standard situation in which the ball is lost to the opponent during a duel or a dribble. The assessment was performed by expert anaylsis coach without bias. A trained coach specializing in match and performance analysis, with extensive education, experience, and expertise, assessed the technical activities.

### Statistical analysis

The sample size was calculated utilizing G*Power 3.1.9.4 software, indicating that a minimum of 16 participants is required, based on an 80% power level (1-β), a significance level of 0.05 (α), and a large effect size (d = 0.8). Data are given as mean ± standard deviation (SD). The Shapiro–Wilk test was employed to assess the normality of the variables, and the findings indicated that they were normally distributed. The gathered data were temporally standardized and analyzed comparatively across the experimental conditions using a one-way analysis of variance (ANOVA) with repeated measurements. Bonferroni Post Hoc analysis was employed to evaluate the differences between the groups. The effect size was evaluated by computing the partial eta-squared value (η2). The significance threshold was established at *p* < 0.05. The data were analyzed using SPSS for Windows version 26 (IBM SPSS Statistics, Chicago, IL, USA). The figure was created using the JASP software.

## Results

Table 2 demonstrates that descriptive statistical information of the participants.

**Table 2:** Descriptive statistical information

Variables	25-min^wup^ X̄ ± SD	15-min^wup^ X̄ ± SD	8-min^wup^ X̄ ± SD
HR Average (beat*mean- 1)	174.38 ± 7.97	179.88 ± 7.76	172.14 ± 9.55
HR Max (beat*mean- 1)	186.08 ± 7.01	191.56 ± 7.43	185.89 ± 7.97
HR Max % (beat*mean- 1)	92.09 ± 3.58	94.81 ± 3.79	92.00 ± 3.95
RPE	4.64 ± 0.83	4.97 ± 0.78	5.16 ± 0.66
Enjoyment	40.50 ± 16.12	41.00 ± 14.27	47.00 ± 11.27
Total Player Load	21.68 ± 3.99	21.63 ± 4.21	19.69 ± 3.96
Total Distance Covered (m)	1150.00 ± 89.14	1171.25 ± 91.79	1120.00 ± 82.14
Average Velocity (km.h)	4.10 ± 0.38	4.33 ± 0.36	4.12 ± 0.38
Maximum Velocity (km.h)	16.76 ± 6.41	15.52 ± 1.73	16. 69 ± 6.48
Succesful Pass	2.56 ± 1.41	10.56 ± 4.11	9.06 ± 2.35
UnSuccesful Pass	0.63 ± 0.72	2.88 ± 1.71	1.63 ± 1.31
Interception	0.69 ± 0.79	2.69 ± 1.62	2.63 ± 1.31
Lost Ball	0.63 ± 0.62	1.94 ± 1.88	3.69 ± 1.66

X̄ mean, *SD* Standart deviation, *Wup* Warm Up, *RPE* rating of perceived exertion

**Fig. 2:**
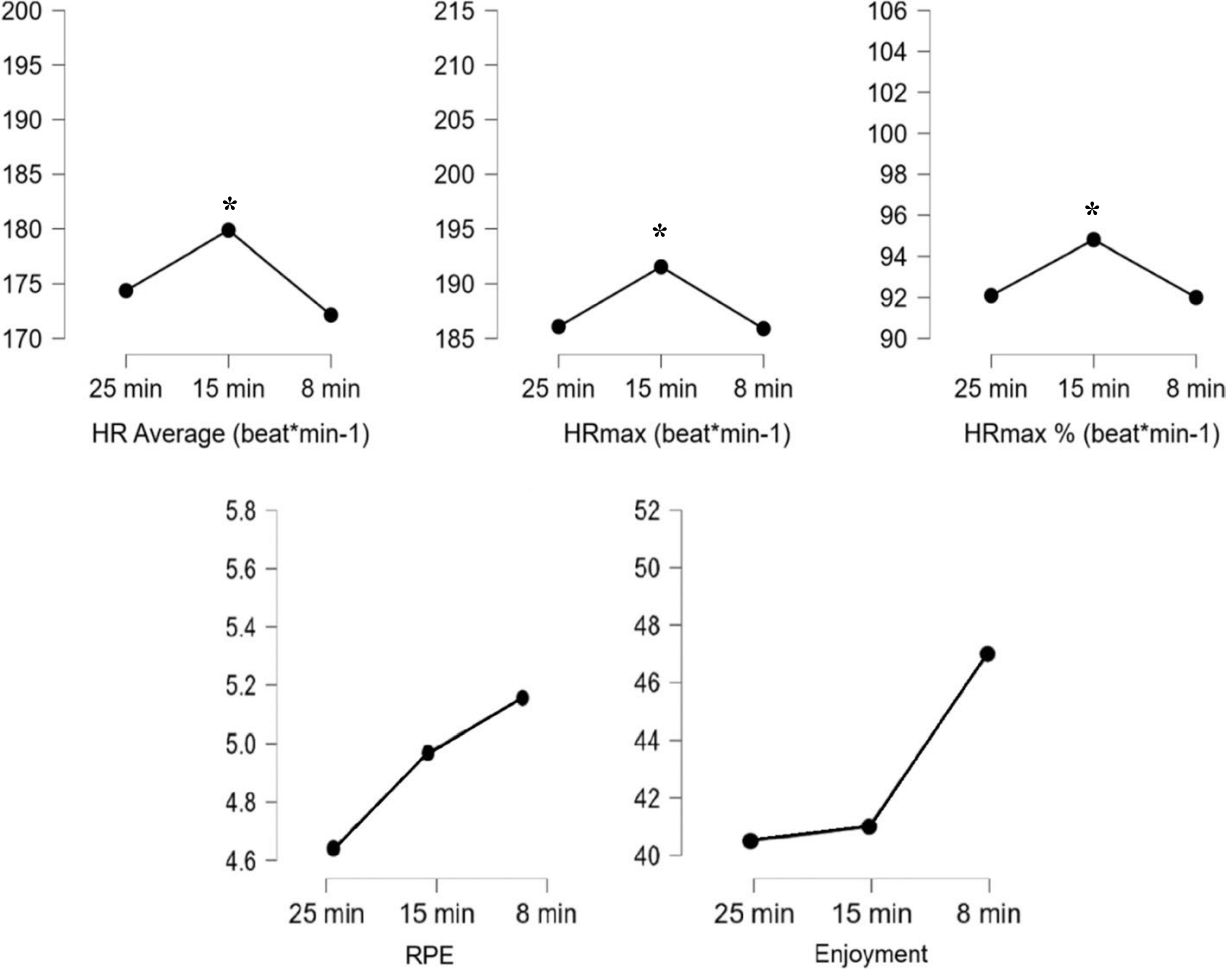
Internal load responses

Figure 2 shows that a repeated-measures ANOVA revealed a statistically significant differences between 25-min^wup^, 15-min^wup^, 8-min^wup^ in HR average (*F* = 5.199, *p* < 0.014 n_p_^2^ = 0.257), HRmax (*F* = 4.660, *p* < 0.019, n_p_^2^ = 0.237), HRmax% (*F* = 4.674, *p* < 0.019, n_p_^2^ = 0.238). Post-hoc tests indicate that 15-min^wup^ duration provides greater increases in heart rate responses compared to 25-min^wup^ and 8-min^wup^ durations. No significant difference was observed between 25-min^wup^, 15-min^wup^, 8-min^wup^ in RPE and enjoyment response (*p* > 0.05).

**Fig. 3:**
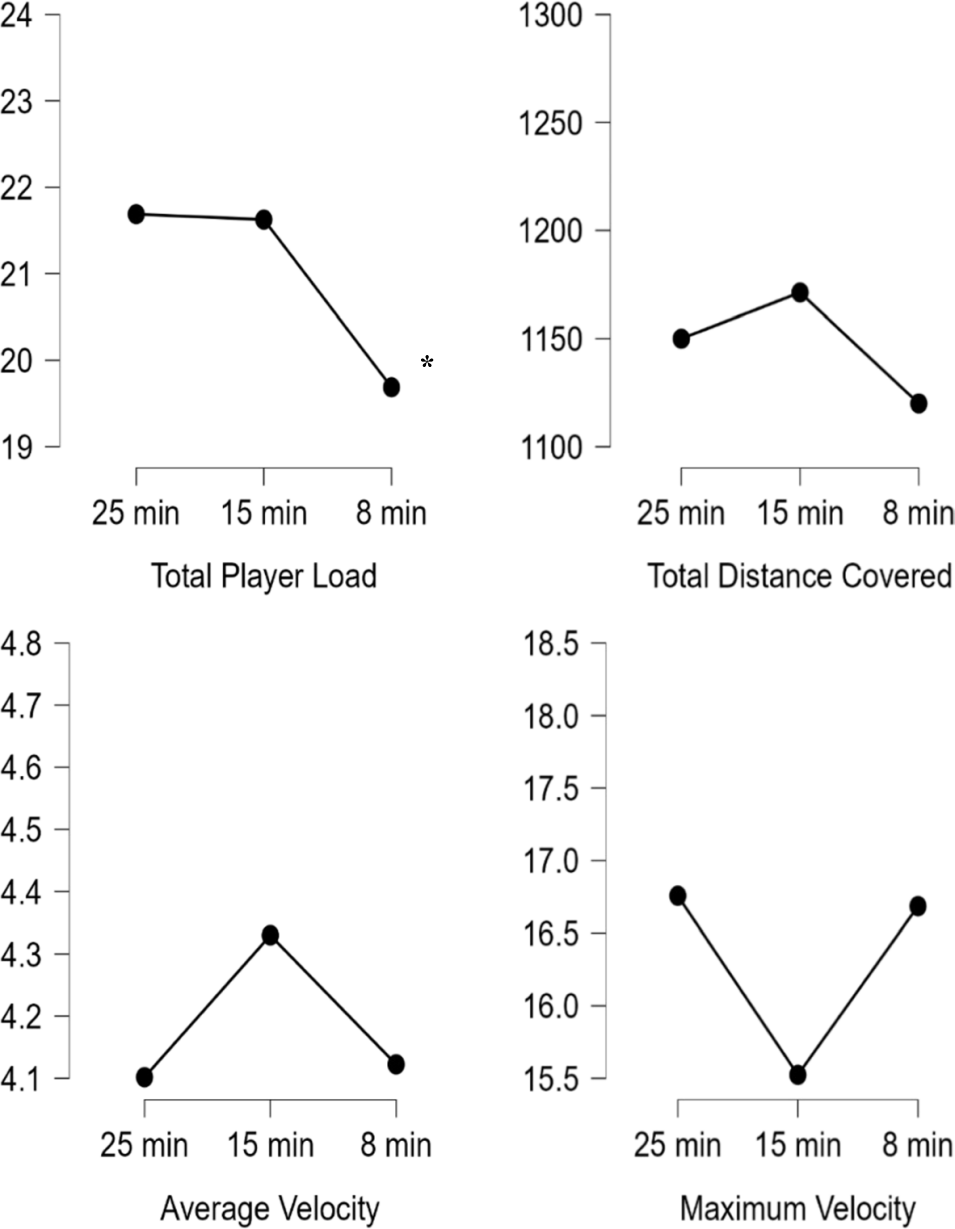
External load response

Figure 3 demonstrates that a repeated-measures ANOVA revealed a statistically significant differences between 25-min^wup^, 15-min^wup^, 8-min^wup^ in total player load (*F* = 20.716 *p* < 0.000, n_p_^2^ = 0.580). Post-hoc tests showed that 8-min^wup^ duration decreased in total player load compared to 25-min^wup^ and 15-min^wup^ durations. No significant difference was observed between 25-min^wup^, 15-min^wup^, 8-min^wup^ in total distance covered, average velocity and maximum velocitiy (*p* > 0.05).

**Fig. 4:**
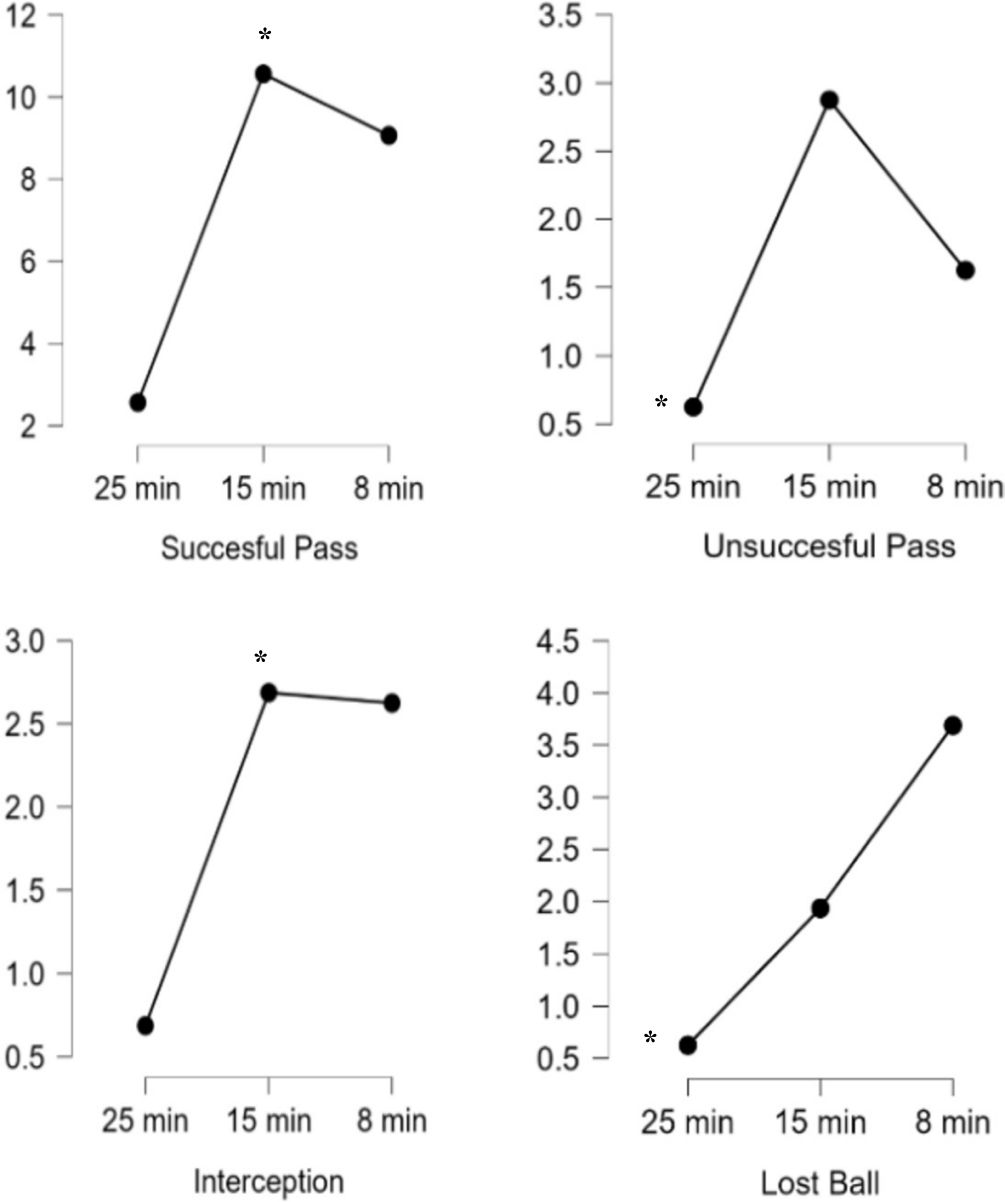
Technical responses

Figure 4 indicate that a repeated-measures ANOVA revealed a statistically significant differences between 25-min^wup^, 15-min^wup^, 8-min^wup^ in succesful pass (*F* = 34.628, *p* < 0.000, n_p_^2^ = 0.698), unsuccesful pass (*F* = 12.039, *p* < 0.001, n_p_^2^ = 0.445), interception (*F* = 13.504, *p* < 0.000, n_p_^2^ = 0.474) and lost ball (*F* = 14.321, *p* < 0.000, n_p_^2^ = 0.488). Post-hoc tests showed that 15-min^wup^ better succesful pass and interception compared to 25-min^wup^ and 8-min^wup^, 25-min^wup^ decreased unsuccesful pass and lost ball compared to 15-min^wup^ and 8-min^wup^.

## Discussion

The findings of this study indicate that varying warm-up durations significantly influence specific technical and physiological performance parameters during SSG in soccer. The 15-min^wup^ emerged as the most effective protocol, resulting in superior successful passes and interceptions compared to both the 8- min^wup^ and 25-min^wup^ protocols. Conversely, the 25-min^wup^ showed no significant advantage in external load, reinforcing that longer warm-ups do not necessarily enhance physical performance. The 8-min^wup^, however, appeared insufficient in activating physiological readiness, resulting in lower player load and less engagement in technical activities.

### Technical performance effects of warm-up duration

The observed improvements in passing and interceptions with the 15-min^wup^ suggest an optimal balance between neuromuscular activation and cognitive readiness. Moderate-duration warm-ups have been shown to increase muscle temperature and motor unit recruitment, leading to enhanced movement precision and reaction time [13, 22]. Additionally, sufficient warm-up duration may improve executive function and decision-making, which are crucial for successful passes and tactical play. This aligns with studies highlighting the importance of cognitive engagement in soccer-specific warm-ups [20, 24].

In contrast, the 25-min^wup^, while beneficial in reducing errors, may lead to early neuromuscular fatigue, diminishing fine motor control required for high-precision technical actions [31, 40]. A prolonged warm-up could lead to excessive muscle activation, making movements less fluid and increasing energy expenditure before gameplay.

#### Physical and physiological impacts on external load

The external load profile further highlights how warm-up duration impacts physical performance. The 15-min^wup^ increased heart rate and player load, supporting a state of optimal physiological readiness without excessive fatigue. Previous studies have demonstrated that short-to-moderate warm-ups effectively prime metabolic systems and neuromuscular responses, leading to enhanced sprinting and agility performance [29].

Conversely, the 8-min^wup^ resulted in lower player load, indicating insufficient cardiovascular and neuromuscular activation. This aligns with research suggesting that warm-ups shorter than 10-min may fail to adequately raise core temperature, thereby limiting muscle elasticity and contraction efficiency [25, 41]. Players who are not physiologically prepared may also engage less in high-intensity actions, explaining the lower external load observed in the 8-min^wup^ protocol.

The 25-min^wup^ showed no significant advantage in external load, reinforcing that longer warm-ups do not necessarily enhance physical performance. Studies indicate that warm-ups exceeding 20-min may induce premature fatigue, reducing sprint efficiency and overall match intensity [40]. Our findings support the notion that a warm-up should be long enough to induce physiological benefits but short enough to avoid energy depletion.

#### Practical applications for coaches and practitioners

These findings provide actionable insights for coaches and practitioners looking to refine warm-up strategies in soccer. A well-structured 15-min^wup^ appears to be the optimal protocol, balancing technical execution and physical preparation without inducing early fatigue. Coaches should prioritize warm-up activities that include the key components outlined in Table 3 to optimize physiological readiness, technical execution, and injury prevention.

**Table 3:** Comprehensive Warm-Up Recommendations

Key Focus	Purpose
Aerobic Activation	Optimize heart rate and muscle temperature
Cognitive & Tactical Drills	Enhance decision-making efficiency
Technical Skill Refinement	Improve passing and interceptions while avoiding excessive fatigue
Dynamic Stretching & Mobility	Increase flexibility, joint mobility, and reduce injury risk
Short-Intensity Acceleration Sprints	Prepare for high-speed movements, improve explosiveness and agility
Small-Sided Game Simulation	Transition into match-like scenarios for tactical and physical readiness
Reactive & Coordination Drills	Develop reaction speed and coordination for better in-game adaptability

For training sessions and matches requiring sustained high-intensity efforts, a 15-min^wup^ ensures players are adequately prepared without compromising performance. Shorter warm-ups may be suitable for low-intensity drills, whereas longer warm-ups may be beneficial for injury prevention in older or high-load athletes but should be carefully monitored to avoid overexertion.

#### Final summary

This study reinforces that warm-up duration plays a critical role in both technical and physiological performance. The 15-min^wup^ strikes the best balance, providing sufficient neuromuscular activation, cognitive readiness, and optimal external load response. Coaches should tailor warm-up strategies based on match demands, player workload, and physiological readiness, ensuring players are primed for peak performance without unnecessary fatigue.

### Limitations and future research

This study has several limitations that should be considered when interpreting the findings. First, the participants were adolescent soccer players with a young age profile and amateur-level experience. As a result, the findings may not be generalizable to players of different age groups, skill levels, or competitive environments. Expanding future research to include a broader range of age demographics, playing positions, and levels of competitive experience would provide a more comprehensive understanding of the effects of warm-up duration.

Second, while this study assessed internal (heart rate, perceived exertion) and external (distance covered, technical skills) load responses, it did not include direct physiological measurements such as core temperature, muscle oxygenation, or metabolic responses. Core temperature is a key physiological marker influencing neuromuscular activation, metabolic efficiency, and fatigue resistance. Future studies should incorporate these measures to provide deeper insights into the underlying physiological mechanisms driving performance differences between warm-up durations.

Third, this study primarily assessed psychological and technical responses during 4-a-side small-sided games with mini-goals. While these metrics are relevant to match-like scenarios, they do not fully capture the physiological and kinematic factors that might also influence performance. Future investigations should incorporate these additional variables to offer a more holistic perspective.

Additionally, the study tested each warm-up condition only once per participant. While this design aimed to reflect real-world warm-up practices in soccer, conducting multiple trials per condition could improve the reliability of the findings. Future studies should consider repeated measures to account for inter-session variability and better isolate the true effects of different warm-up durations on performance outcomes.

Lastly, the focus of this study was on immediate responses during and immediately after the small-sided games. The long-term impacts of varying warm-up durations, such as their effects on injury prevention, performance sustainability, and recovery, remain unexplored. Addressing these gaps in future research would help establish evidence-based guidelines for optimal warm-up strategies in soccer and other team sports.

## Conclusions

In conclusion, our study adds to the growing body of evidence advocating for tailored warm-up durations in soccer. The 15-min^wup^ duration emerged as an optimal protocol, aligning well with physiological, technical, and psychological findings while offering practical implications for efficient training and match preparation. Given the importance of optimizing warm-up strategies in soccer, our findings suggest that a well-structured 15-min^wup^ can enhance technical performance and physiological readiness while minimizing unnecessary fatigue. This insight is particularly relevant for coaches and practitioners aiming to refine pre-match and training routines to maximize player efficiency and performance in small-sided games and match scenarios.

## Data Availability

The dataset supporting the conclusions of this article is included in the article.
